# Mono-allelic retrotransposon insertion addresses epigenetic transcriptional repression in human genome

**DOI:** 10.1186/1423-0127-19-13

**Published:** 2012-02-02

**Authors:** Hyang-Min Byun, Kyu Heo, Kasey J Mitchell, Allen S Yang

**Affiliations:** 1 Jane Anne Nohl Division of Hematology, Norris Comprehensive Cancer Center, Keck School of Medicine, University of Southern California, Los Angeles, California, USA; 2 Department of Biochemistry and Molecular Biology, Norris Comprehensive Cancer Center, University of Southern California, Los Angeles, California, USA; 3 Department of Environmental Health, Harvard School of Public Health, Boston, Massachusetts, USA; 4 Research Center, Dongnam Institute of Radiological and Medical Sciences, 40 Jwadong-gil, Jangan-eup, Gijang-gun, Busan 619-953, Republic of Korea; 5 Amgen, Inc., 1 Amgen Center Drive, MS 38-2-A, Thousand Oaks, California 91320-1799, USA

**Keywords:** Epigenetics, Retrotransposons, Long interspersed elements, *Alu*

## Abstract

**Background:**

Retrotransposons have been extensively studied in plants and animals and have been shown to have an impact on human genome dynamics and evolution. Their ability to move within genomes gives retrotransposons to affect genome instability.

**Methods:**

we examined the polymorphic inserted *Alu*Ya5, evolutionary young *Alu*, in the progesterone receptor gene to determine the effects of *Alu *insertion on molecular environment. We used mono-allelic inserted cell lines which carry both *Alu*-present and *Alu*-absent alleles. To determine the epigenetic change and gene expression, we performed restriction enzyme digestion, Pyrosequencing, and Chromatin Immunoprecipitation.

**Results:**

We observed that the polymorphic insertion of evolutionally young *Alu *causes increasing levels of DNA methylation in the surrounding genomic area and generates inactive histone tail modifications. Consequently the *Alu *insertion deleteriously inactivates the neighboring gene expression.

**Conclusion:**

The mono-allelic *Alu *insertion cell line clearly showed that polymorphic inserted repetitive elements cause the inactivation of neighboring gene expression, bringing aberrant epigenetic changes.

## Background

Retrotransposons have been extensively studied in plants and animals and have been shown to have an impact on human genome dynamics and evolution. About 42% of the human genome contains retrotransposons while DNA transposons account for around 2-3% [1-3]. According to the 2001 analysis, which has been confirmed overall by the 2004 update (International Human Genome Sequencing Consortium 2004), short interspersed elements (SINEs), such as *Alu *or SINE-R/VNTR/*Alu *(SVA), account for 13%, Long interspersed elements [LINE-1(L1)] for 20%, and long-terminal repeat (LTR) retrotransposons, such as endogenous retrovirus (ERV), for 8%, respectively, of the sequenced human genome. The retrotransposons increase their copy number by retrotransposition via RNA. Attempted or successful retrotranspositions carry a high risk of eliciting chromosome breaks, deletions, translocations, and recombinations [4]. It is estimated that there is one *Alu *retrotransposon insertion every 21 births [5] during gametogenesis, transferring the retrotransposon's genetic information to the next generation [6,7]. These retrotransposition events are likely to change the activity of genes at the insertion site, including increased or decreased transcriptional activity. In some cases, this alteration of gene expression causes the development of several diseases or cancers [8]. DNA methylation on the retrotransposon is thought to be the mechanism that controls the retrotransposition rate. Recent vast numbers of publications uniformly address that complex disease, cancer, aging, and environmental challenges are associated with aberrant retrotransposon DNA methylation.

In fact, not all retrotransposons have the capability to retrotranspose to other genomic locations. Currently, most L1s are inactive and cannot retrotranspose to new genomic locations [9], while a small number of human specific L1 (L1HS) elements remain retrotransposition competent. Retrotransposons seeded in the human genome several million years ago and have many subfamilies defined by distinct patterns of diagnostic base substitutions. Subfamilies may be classified as young, intermediate or old, reflecting the time since the start of retroposition by their members. The expansion of *Alu *subfamilies (Yc1, Ya5, Ya2, Yb9, Yb8, Y, Sg1, Sx, and J; young to old, respectively) is superimposed on primate evolution. The evolutionally young L1, *Alu*, and SVA are currently able to transpose in the human genome, hence the ongoing retrotranspositional insertions of the youngest subfamilies are not yet fixed in the human genome and represent polymorphic loci [10]. Some polymorphic insertions are known to be responsible for more than 30 human genetic diseases [11-13]. A genetic polymorphism names as PROGINS has been identified in the progesterone receptor (*PGR*) gene with insertion of *Alu *subfamily [14]. The correlations of *Alu *insertion polymorphism on *PGR *gene are associated with endometriosis [15,16], ovarian cancer with diethylstilbestrol exposure [17], breast cancer [18], and obesity [19]. Insertional polymorphic retrotransposons are often observed in a mono-allelic fashion, meaning retrotransposons are inserted into only one of the alleles in individuals. For instance, in chromosome 11, the *PGR *gene has a newly inserted *Alu*Ya5 subfamily between exon 5 and 6. In this study, we examine DNA methylation and histone modification of the locus which occurred mono-allelic young *Alu, Alu*Ya5 insertions and address the direct effect of retrotransposon in controlling gene expression.

## Methods

### Nucleic acid isolation and bisulfite treatment

Genomic DNA was isolated by standard proteinase K digestion and phenol-chloroform extraction [20]. Total RNA was collected and extracted from cultured cells with the RNeasy Protect minikit (QIAGEN Inc., Valencia, CA) according to the manufacturer's recommended protocol. Reverse transcription was performed by using the first strand cDNA synthesis kit (NEB, Beverly, MA, USA). Bisulfite modification of genomic DNA has been described previously [21]. PCR primer sequences for *Alu *polymorphism with genomic DNA were forward: TTGAGTAAAGCCTCTAAAAT and reverse: TTCTTGCTAAATGTCTGTT, and with bisulfite DNA were forward: GAAATTTGAAGGAAATAAATATTAGTGT and reverse: CATTTAATTATCCAAAAATATTTTCTTACTAA.

### Quantitation of allele-specific gene expression by Pyrosequencing

PCR products from genomic DNA or cDNA were used for Pyrosequencing analysis as previously described [21]. Briefly, the PCR product of each gene was used for individual sequencing reactions. Streptavidin-Sepharose beads (Amersham Biosciences) and a Vacuum Prep Tool (Biotage AB) were used to purify the single-stranded biotinylated PCR products according to the manufacturer's recommendation. The appropriate sequencing primer was annealed to the purified PCR product and used for a Pyrosequencing reaction using the PSQ 96HS system (Biotage). Raw data were analyzed with the allele quantitation algorithm using the PSQ 96 HS software. PCR primer sequences for *Alu *polymorphism by Pyrosequencing were forward: TTTTCGAAACTTACATATTGA, reverse biotin labeled: TTTAGTATTAGATCAGGTGC, and sequencing primer: GATCCTACAAACA. For allele-specific expression, forward primers: TAGTCAAGTGGTCTAAATCATTGC, reverse biotin labeled: TTTAGTATTAGATCAGGTGC, and sequencing primer: GATCCTACAAACA. To validate DNA methylation detection by Pyrosequencing, we designed control oligo for 100% DNA methylation (PSQ-C oligo: 5'- TATTAGATCGACGGGAACAAACGTTGAATTC -3') and 0% DNA methylation (PSQ-T oligo: 5'- TATTAGATCAACGGGAACAAACGTTGAATTC -3'). The sequencing primer for control oligo is 5'- CAACGTTTGTTCCCGT -3'. We mixed PSQ-C oligo (or PSQ-T oligo) with sequencing oligo in PyroMark Annealing Buffer (QIAGEN Inc., Valencia, CA) and performed Pyrosequencing with sequencing entry C/TGATC.

### Chromatin Immunoprecipitation

ChIP assays were performed as described previously [22]. Briefly, 25 μg crosslinked protein-DNA complexes were immunoprecipitated using two different histone modification antibodies (H3K9ac, H3K9me3: Millipore) and eluted. Eluted DNA fragments were amplified by PCR. PCR primer sequences for the multiplex PCR reaction were L1 forward: GCCTTGCAGTTTGATCTCAG and reverse: GACGGGTGATTTCTGCATTT, *Alu*Y8 forward: GTGGCTCACGCCTGTAATCCCAGC and reverse: GTCGCCCAGGCCGGACTGCG, and *Alu*J forward: TGGCTCACGCCTGTAATCCCAG and reverse: GCCTCGACCTCCCGGGCTCAAGCG. Analyzing density of gel bands was performed using ImageJ which is a public domain Java image processing program (http://rsb.info.nih.gov/ij/).

## Results

### Screening of *Alu*Ya5 insertional polymorphisms in cell lines

**Figure 1:**
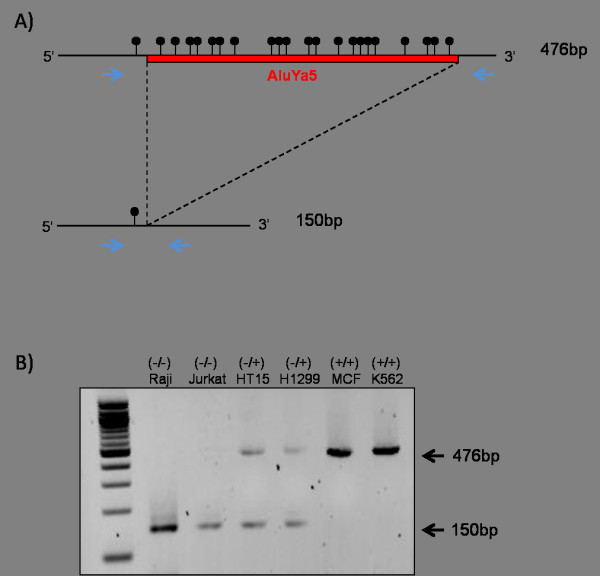
**Schematic diagram and genotyping of AluYa5 insertion**. A) Schematic of mono-allelic *Alu*Ya5 insertion in Chromosome 11. The size of *Alu*-present allele is 476 bp and *Alu*-absent allele is 150 bp. The red line is *Alu*Ya5 inserted in human genome. Black circles represent CpG sites. The location of PCR primers are represented in blue arrows. B) The genotyping of *Alu *insertion. The genomic DNA from six cell lines, Raji, Jurkat, HT15, H1299, MCF, and K562, are amplified with PCR primer marked above. Single 150 bp bands are generated from bi-allelic *Alu*-absent cell lines, 150 bp and 476 bp are from mono-allelic inserted cell lines, and single 476 bp are produced from bi-allelic inserted cell line.

To find insertional polymorphic retrotransposons, we screened Raji, Jurkat, HT15, H1299, MCF, and K562 cell lines using the primer sets listed in the Methods section. The primers flanked the newly inserted retrotransposon *Alu*Ya5 in chr11:100,911,358-100,912,065 locus (Assembly: hg19), thus presence of *Alu *insertion could be distinguished by length of PCR amplicon. The PCR amplicon with fully inserted *Alu *generates a 476 bp product, while the amplicon without *Alu *insertion produces a 150 bp product. Among the cell lines we tested, HT15 and H1299 showed two different sizes of bands after PCR amplification, indicating *Alu *has inserted in only one allele of the genome locus (Figure 1). MCF and K562 showed insertion of *Alu *into both alleles (476 bp products). Raji and Jurkat cell lines, however, did not carry an *Alu *insertion in either allele (150 bp products).

### *Alu *insertion dependent DNA cytosine methylation

**Figure 2:**
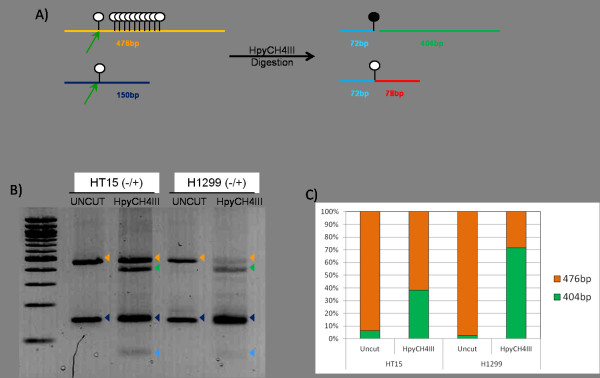
***Alu *insertion dependent restriction enzyme digestion**. A) Predicted size and cutting site after restriction enzyme digestion. B) Agarose gel electrophoresis and C) density of gel bands. Bisulfite-PCR products from mono-allelic *Alu *inserted cell lines, HT15 and H1299, are digested with HpyCH4III restriction enzyme. 'Uncut' represent a pre-digestion of PCR products which show the intact *Alu*-present (orange line and arrow heads) and *Alu*-absent (dark blue line and arrow heads). 'HpyCH4III' represent a post-digestion of PCR products which generate 72 bp bands (light blue lines and arrow heads) and either 404 bp (green lines and arrow heads) or 78 bp (red lines and arrow heads), depends on the DNA methylation status in the CpG site. The density of the gel band from *Alu*-present allele was measured.

In order to examine the retrotransposon-derived DNA methylation spreading theory [23], we determined DNA methylation status on the *Alu*-present and *Alu*-absent alleles, using the mono-allelic inserted cell lines HT15 and H1299. The PCR amplicon with bisulfite treated DNA was digested with the restriction enzyme HpyCH4III, which cut the 5'..ACNGT..3' region located on the PCR amplicon sequence in only the methylated allele (Figure 2A). Both mono-allelic *Alu *inserted cell lines, HT15 and H1299, showed partial digestion of only the *Alu*-present allele, indicating DNA methylation exists in only the *Alu *inserted allele (Figure 2B). The *Alu *inserted allele in the H1299 cell line showed slightly more methylation than the *Alu *inserted allele in the HT15 cell line (Figure 2C). We did not observe digestion of the *Alu*-absent allele.

### *Alu *insertion derived inactive histone modification

**Figure 3:**
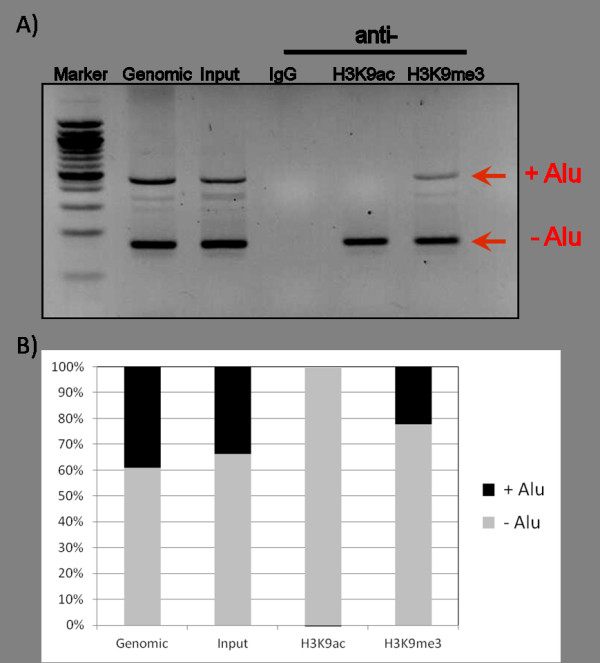
**Chromatin Immunoprecipitation assay to assess differential histone modifications on *Alu*-absent or *Alu*-present allele**. Representative gel showing chromatin preparations from immunoprecipitated with anti-IgG (lanes 4), anti-H3K9ac (lane 5), anti-H3K9me3 (lane 6), or the negative control non-immune serum (lanes 3). Lane 2 is non-immunoprecipitated DNA. Lane 1 is DNA size marker (A). The density of the gel band from *Alu*-present and *Alu*-absent allele was measured (B). '+ *Alu*' is the *Alu*-present allele and '- *Alu*' represent the *Alu*-absent allele.

**Figure 4:**
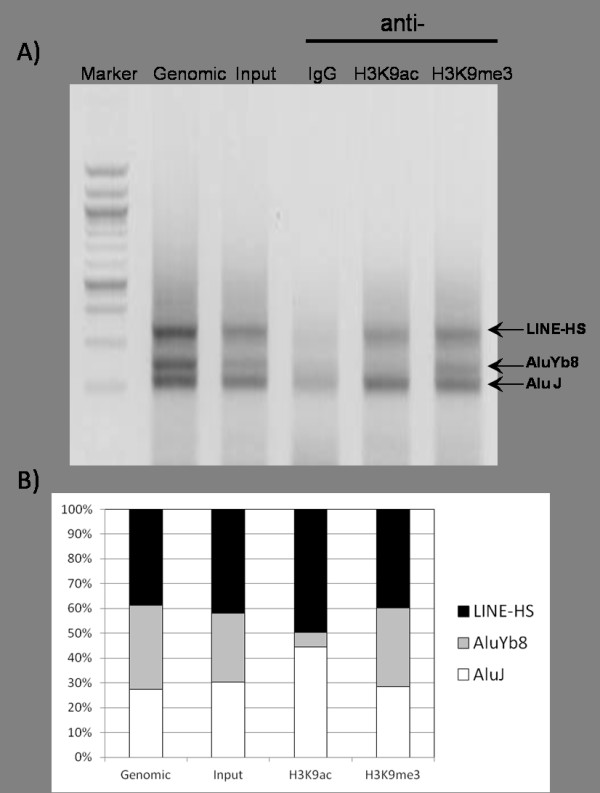
**Chromatin Immunoprecipitation assay to assess differential histone modifications of global L1HS, young *Alu; Alu*Yb8 and old *Alu; Alu*J**. Representative gel showing chromatin preparations from immunoprecipitated with anti-IgG (lanes 4), anti-H3K9ac (lane 5), anti-H3K9me3 (lane 6), or the negative control non-immune serum (lanes 3). Lane 2 is non-immunoprecipitated DNA. Lane 1 is DNA size marker (A). The density of the gel band from L1HS, *Alu*Yb8, and *Alu*J was measured (B).

To determine whether *Alu *insertion causes histone tail modifications, we performed ChIP-PCR with two histone modification antibodies against H3K9ac or H3K9me3. Acetylation at Lys-9 on histone H3 (H3K9ac) is an active chromatin marker and often associated with positive gene expression; conversely, methylation at Lys-9 on histone H3 (H3K9me3) is an inactive chromatin marker and correlated with repressed gene expression [24]. After chromatin immunoprecipitation with the two antibodies for active or repressive histone markers, followed by PCR amplification, we observed differential histone modification between *Alu*-present and *Alu*-absent alleles. The active marker H3K9ac is present in only the Alu-absent allele; however, the inactive histone marker H3K9me3 exists in both allele of the genome locus (Figure 3). This difference in histone modification has only happened in young *Alu *subfamilies, not all *Alu *subfamilies. ChIP coupled with PCR amplification of *Alu*J, *Alu*Yb8, and L1HS showed different distributions of histone modifications. *Alu*J, the oldest *Alu *subfamily, co-located with both the active marker H3K9ac and the inactive marker H3K9me3. However, the young *Alu *subfamily *Alu*Yb8 had at least eight times more inactive histone marker H3K9me3. In addition, human-specific L1HS did not show a different distribution of active or inactive histone markers (Figure 4).

### Gene expression repressed by *Alu *insertion in the genome

**Figure 5:**
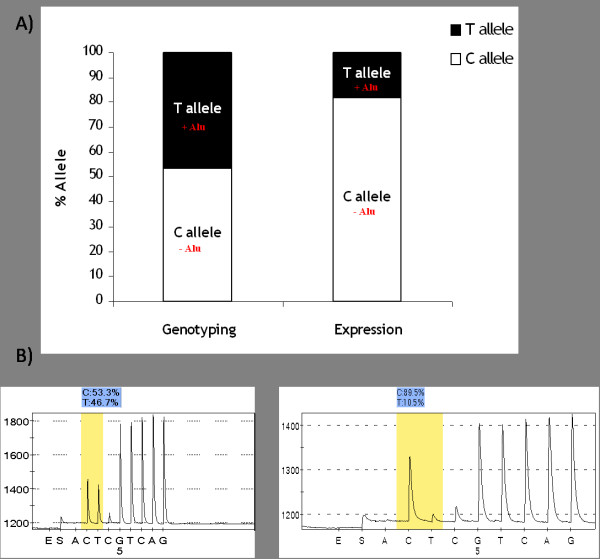
**Differential level of *PGR *gene expression on *Alu*-absent or -present allele**. A) PCR combined with Pyrosequencing shows similar proportion of C (*Alu*-absent; 53.3%) and T allele (*Alu*-present; 46.7%) in genomic DNA. Reverse transcription-PCR coupled with Pyrosequencing shows different proportion of C (*Alu*-absent; 89.5%) and T allele (*Alu*-present; 10.5%). B) Representative program of genotyping and gene expression.

To examine differential gene expression in *Alu*-present and *Alu*-absent allele, we developed an allele-specific gene expression detection method using Pyrosequencing. To distinguish between the two alleles, we genotyped the single nucleotide polymorphism (SNP) at chr11:100921952-100922452 (2009 (GRCh37/hg19) assembly), reference SNP ID number is rs1042839, since this SNP is correlated with occurrence of *Alu *insertion [25,26]. To confirm this co-existence, we genotyped this SNP in the six cell lines we worked with and compared with their *Alu *insertion statuses (Table 1). Hetero *Alu *inserted cell lines HT15 and H1299 showed heterozygote C/T, *Alu*-absent cell lines Hep3B2 and HL-60 had a C/C genotype, and *Alu*-present cell lines MCF and K562 had a T/T genotype. We confirmed that the T allele co-exists with *Alu *insertion, while the C allele co-exists with the absence of *Alu *insertion in hetero *Alu*-inserted cell lines. Next, we used this SNP to identify *Alu*-inserted alleles for allele-specific gene expression detection in a Pyrosequencing reaction. After reverse transcription-PCR with mRNA from the hetero *Alu*-inserted cell line H1299, we amplified the locus flanking the SNP to detect each allelic gene expression level (Figure 5). Surprisingly, we observed unequal gene expression levels between *Alu*-present and *Alu*-absent alleles, 10.5% and 89.5% respectively, having an equal distribution of both alleles in the genome (46.7% of *Alu*-present allele vs 53.3% of *Alu*-absent alleles with genotyping data). Thus the presence of *Alu *in the gene body repressed gene expression at the allele containing the *Alu *element.

**Table 1:** *Alu *insertion and genotyping in cell lines

	Hep3B2	HL-60	HT15	H1299	MCF	K562
***Alu***	-/-	-/-	-/+	-/+	+/+	+/+

**SNP**	CC	CC	TC	TC	TT	TT

## Discussion

**Figure 6:**
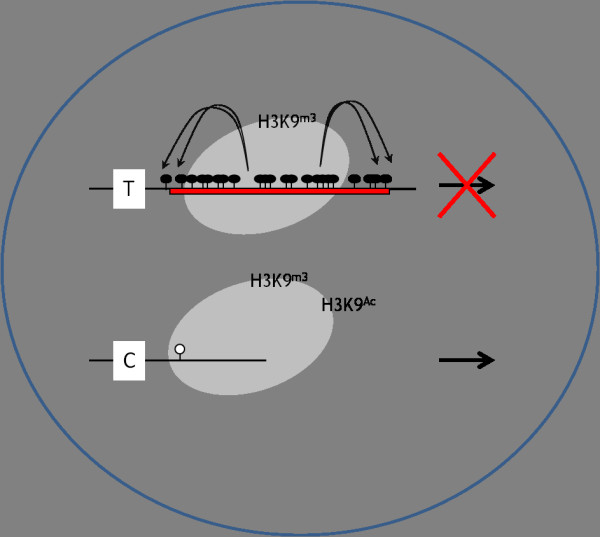
**Summary of molecular signature on mono-allelic *Alu *insertion**. *Alu*-present allele has cytosine methylation in neighbor CpG and repressive histone modification. *Alu*-absent allele shows no CpG methylation in near CpG site and has additionally active histone modification, hence show active gene expression.

We examined the polymorphic inserted young *Alu, Alu*Ya5, in the *PGR *gene to determine the effects of *Alu *insertion on the near gene environment. We used mono-allelic inserted cell lines which carry both *Alu*-present and *Alu*-absent alleles. We observed that the polymorphic insertion of evolutionally young *Alu *causes increasing levels of DNA methylation in the surrounding genomic area and generates inactive histone tail modifications. Consequently, the *Alu *insertion deleteriously inactivates the neighboring gene expression (Figure 6).

It is a novel approach to address the cis-effects of retrotransposons or retrotransposition in neighboring genomic structures using a mono-allelic inserted young *Alu *subfamily. These effects were observed in a single cell line system, and virtually all conditions at the particular locus are the same; the only difference being the presence or absence of a retrotransposon insertion. Thus this system bypasses many concerns about experimental artifacts being solely responsible for deducing the function of retrotransposons in the genome.

Generally, our results agree with previous reports that retrotransposons may repress gene expression through an epigenetic mechanism. Our study strongly supports the observations that young active retrotransposons insert in areas that lack cytosine methylation. Retrotransposons spread DNA methylation into neighboring regions, generating repressive histone modifications. It causes a significant inactivation of gene expression. Hollister et al. reported the correlation of transposable elements and gene silencing; however the caveat was that the data do not show whether repetitive elements tend to preferentially insert near lowly expressed genes or whether the insertion of repetitive elements causes the low gene expression [27]. However, our mono-allelic inserted cell line system clearly showed that repetitive elements cause the inactivation of neighboring gene expression.

It has been estimated that approximately one out of every 21 births, 212 births, and 916 births has a new insertion of *Alu*, L1, and SVA retrotransposition, respectively [10]. Thus there is a great deal of retrotransposition in the current human genome. It has been know that evolutionally young repetitive elements have the capability to retrotranspose to other genomic locations. In our study, the inactive histone modification solely existed in the young *Alu *subfamily and disappeared in the old *Alu *J subfamily. Coincidently, global *Alu *J has less DNA methylation than the young and active *Alu *Y family (data not shown). Hence this epigenetic difference may promote the mechanism that facilitates transposon mobility. However, what triggers this phenomenon is still not clear, though environmental cues are believed to be responsible for promoting movement of DNA transposons and retrotransposons. DNA methylation on retrotransposons is thought to be an intermediate of the retrotransposition mechanism. We have observed aberrant cytosine methylation patterns on retrotransposons with environmental challenges, but do not know what causes these events or what consequences follow them. Based on the observation of our data, aberrant cytosine methylation on retrotransposons caused by environmental challenges may trigger retrotransposon mobility, slowly reshaping human genome. In the future, it will be necessary to understand the function of other types of retrotransposons of different ages in order to finally resolve the meaning of this aberrant epigenetic phenomenon driven by environmental challenge.

## Conclusions

The mono-allelic Alu insertion cell line clearly showed that polymorphic inserted repetitive elements cause the inactivation of neighboring gene expression, bringing aberrant epigenetic changes.

## Competing interests

The authors declare that they have no competing interests.

## Authors' contributions

HMB designed the experiment. KH performed Chromatin immunocytochemistry and HMB performed rest of experiments. HMB, KH, KM, ASY prepared the manuscript and ASY oversaw the research. All authors have read and approved the final manuscript.

## References

[B1] SanMiguel P Gaut BS Tikhonov A Nakajima Y Bennetzen JL The paleontology of intergene retrotransposons of maize Nat Genet 1998 20 43 45 10.1038/1695 9731528

[B2] Li W Zhang P Fellers JP Friebe B Gill BS Sequence composition, organization, and evolution of the core Triticeae genome Plant J 2004 40 500 511 10.1111/j.1365-313X.2004.02228.x 15500466

[B3] Lander ES Linton LM Birren B Initial sequencing and analysis of the human genome Nature 2001 409 860 921 10.1038/35057062 11237011

[B4] Symer DE Connelly C Szak ST Caputo EM Cost GJ Parmigiani G Boeke JD Human l1 retrotransposition is associated with genetic instability in vivo Cell 2002 110 327 338 10.1016/S0092-8674(02)00839-5 12176320

[B5] Xing J Zhang Y Han K Salem AH Sen SK Huff CD Zhou Q Kirkness EF Levy S Batzer MA Jorde LB Mobile elements create structural variation: analysis of a complete human genome Genome Res 2009 19 1516 1526 10.1101/gr.091827.109 19439515 PMC2752133

[B6] Branciforte D Martin SL Developmental and cell type specificity of LINE-1 expression in mouse testis: implications for transposition Mol Cell Biol 1994 14 2584 2592 10.1128/MCB.14.4.2584 8139560 PMC358626

[B7] Ergun S Buschmann C Heukeshoven J Dammann K Schnieders F Lauke H Chalajour F Kilic N Stratling WH Schumann GG Cell type-specific expression of LINE-1 open reading frames 1 and 2 in fetal and adult human tissues J Biol Chem 2004 279 27753 27763 10.1074/jbc.M312985200 15056671

[B8] Wolff EM Byun HM Han HF Sharma S Nichols PW Siegmund KD Yang AS Jones PA Liang G Hypomethylation of a LINE-1 promoter activates an alternate transcript of the MET oncogene in bladders with cancer PLoS Genet 2010 6 e1000917 10.1371/journal.pgen.1000917 20421991 PMC2858672

[B9] Brouha B Schustak J Badge RM Lutz-Prigge S Farley AH Moran JV Kazazian HH Jr Hot L1s account for the bulk of retrotransposition in the human population Proc Natl Acad Sci USA 2003 100 5280 5285 10.1073/pnas.0831042100 12682288 PMC154336

[B10] Li X Scaringe WA Hill KA Roberts S Mengos A Careri D Pinto MT Kasper CK Sommer SS Frequency of recent retrotransposition events in the human factor IX gene Hum Mutat 2001 17 511 519 10.1002/humu.1134 11385709

[B11] Deininger PL Batzer MA Alu repeats and human disease Mol Genet Metab 1999 67 183 193 10.1006/mgme.1999.2864 10381326

[B12] Miki Y Retrotransposal integration of mobile genetic elements in human diseases J Hum Genet 1998 43 77 84 10.1007/s100380050045 9621510

[B13] Ostertag EM Kazazian HH Jr Biology of mammalian L1 retrotransposons Annu Rev Genet 2001 35 501 538 10.1146/annurev.genet.35.102401.091032 11700292

[B14] Rowe SM Coughlan SJ McKenna NJ Garrett E Kieback DG Carney DN Headon DR Ovarian carcinoma-associated TaqI restriction fragment length polymorphism in intron G of the progesterone receptor gene is due to an Alu sequence insertion Cancer Res 1995 55 2743 2745 7796397

[B15] Wieser F Schneeberger C Tong D Tempfer C Huber JC Wenzl R PROGINS receptor gene polymorphism is associated with endometriosis Fertil Steril 2002 77 309 312 10.1016/S0015-0282(01)02984-3 11821088

[B16] Lattuada D Somigliana E Vigano P Candiani M Pardi G Di Blasio AM Genetics of endometriosis: a role for the progesterone receptor gene polymorphism PROGINS? Clin Endocrinol (Oxf) 2004 61 190 194 10.1111/j.1365-2265.2004.02076.x 15272913

[B17] Engehausen DG Schrott KM PROGINS polymorphism of progesterone receptor is increased in female offspring with maternal exposure to diethylstilbestrol Anticancer Res 2000 20 5145 5149 11326686

[B18] Wang-Gohrke S Chang-Claude J Becher H Kieback DG Runnebaum IB Progesterone receptor gene polymorphism is associated with decreased risk for breast cancer by age 50 Cancer Res 2000 60 2348 2350 10811106

[B19] Wasserman L Flatt SW Natarajan L Laughlin G Matusalem M Faerber S Rock CL Barrett-Connor E Pierce JP Correlates of obesity in postmenopausal women with breast cancer: comparison of genetic, demographic, disease-related, life history and dietary factors Int J Obes Relat Metab Disord 2004 28 49 56 10.1038/sj.ijo.0802481 14557830

[B20] Byun HM Wong HL Birnstein EA Wolff EM Liang G Yang AS Examination of IGF2 and H19 loss of imprinting in bladder cancer Cancer Res 2007 67 10753 10758 10.1158/0008-5472.CAN-07-0329 18006818

[B21] Byun HM Siegmund KD Pan F Weisenberger DJ Kanel G Laird PW Yang AS Epigenetic profiling of somatic tissues from human autopsy specimens identifies tissue- and individual-specific DNA methylation patterns Hum Mol Genet 2009 18 4808 4817 10.1093/hmg/ddp445 19776032 PMC4481584

[B22] Nishioka K Chuikov S Sarma K Erdjument-Bromage H Allis CD Tempst P Reinberg D Set9, a novel histone H3 methyltransferase that facilitates transcription by precluding histone tail modifications required for heterochromatin formation Genes Dev 2002 16 479 489 10.1101/gad.967202 11850410 PMC155346

[B23] Arnaud P Goubely C Pelissier T Deragon JM SINE retroposons can be used in vivo as nucleation centers for de novo methylation Mol Cell Biol 2000 20 3434 3441 10.1128/MCB.20.10.3434-3441.2000 10779333 PMC85636

[B24] Jenuwein T Allis CD Translating the histone code Science 2001 293 1074 1080 10.1126/science.1063127 11498575

[B25] Agoulnik IU Tong XW Fischer DC Korner K Atkinson NE Edwards DP Headon DR Weigel NL Kieback DG A germline variation in the progesterone receptor gene increases transcriptional activity and may modify ovarian cancer risk J Clin Endocrinol Metab 2004 89 6340 6347 10.1210/jc.2004-0114 15579801

[B26] De Vivo I Huggins GS Hankinson SE Lescault PJ Boezen M Colditz GA Hunter DJ A functional polymorphism in the promoter of the progesterone receptor gene associated with endometrial cancer risk Proc Natl Acad Sci USA 2002 99 12263 12268 10.1073/pnas.192172299 12218173 PMC129433

[B27] Hollister JD Gaut BS Epigenetic silencing of transposable elements: a trade-off between reduced transposition and deleterious effects on neighboring gene expression Genome Res 2009 19 1419 1428 10.1101/gr.091678.109 19478138 PMC2720190

